# Cryopreserved vitamin D_3_-tolerogenic dendritic cells pulsed with autoantigens as a potential therapy for multiple sclerosis patients

**DOI:** 10.1186/s12974-016-0584-9

**Published:** 2016-05-20

**Authors:** María José Mansilla, Raian Contreras-Cardone, Juan Navarro-Barriuso, Nathalie Cools, Zwi Berneman, Cristina Ramo-Tello, Eva María Martínez-Cáceres

**Affiliations:** Division of Immunology, Germans Trias i Pujol University Hospital and Research Institute, Campus Can Ruti, Badalona, Spain; Department of Cellular Biology, Physiology and Immunology, Universitat Autònoma de Barcelona, 08193 Bellaterra (Cerdanyola del Vallès), Spain; Laboratory of Experimental Hematology, Vaccine and Infectious Disease Institute (VAXINFECTIO), Antwerp University Hospital, Faculty of Medicine and Health Sciences, University of Antwerp, 2610 Wilrijk, Belgium; Multiple Sclerosis Unit, Department of Neurosciences, Hospital Universitari Germans Trias i Pujol, Badalona, Spain

**Keywords:** Tolerogenic dendritic cells, Multiple sclerosis, EAE, Treg, Breg, NK cells, NKT cells

## Abstract

**Background:**

Tolerogenic dendritic cells (tolDC) have been postulated as a potent immunoregulatory therapy for autoimmune diseases such as multiple sclerosis (MS). In a previous study, we demonstrated that the administration of antigen-specific vitamin D_3_ (vitD3) tolDC in mice showing clinical signs of experimental autoimmune encephalomyelitis (EAE; the animal model of MS) resulted in abrogation of disease progression. With the purpose to translate this beneficial therapy to the clinics, we have investigated the effectivity of vitD3-frozen antigen-specific tolDC pulsed with myelin oligodendrocyte glycoprotein 40-55 peptide (f-tolDC-MOG) since it would reduce the cost, functional variability and number of leukapheresis to perform to the patients.

**Methods:**

Mice showing EAE clinical signs were treated with repetitive doses of f-tolDC-MOG. Tolerogenic mechanisms induced by the therapy were analysed by flow cytometry and T cell proliferation assays.

**Results:**

Treatment with f-tolDC-MOG was effective in ameliorating clinical signs of mice with EAE, inhibiting antigen-specific reactivity and inducing Treg. In addition, the long-term treatment was well tolerated and leading to a prolonged maintenance of tolerogenicity mediated by induction of Breg, reduction of NK cells and activation of immunoregulatory NKT cells.

**Conclusions:**

The outcomes of this study show that the use of antigen-specific f-tolDC promotes multiple and potent tolerogenic mechanisms. Moreover, these cells can be kept frozen maintaining their tolerogenic properties, which is a relevant step for their translation to the clinic. Altogether, vitD3 f-tolDC-MOG is a potential strategy to arrest the autoimmune destruction in MS patients.

## Background

Multiple sclerosis (MS) is a complex chronic, inflammatory and demyelinating disease affecting the central nervous system (CNS) [1, 2]. Studies performed in patients and animal models support that MS pathogenesis is mediated by autoreactive T cells recognizing myelin peptides [3, 4]. Different types of unspecific immunomodulatory or immunosuppressive drugs, as well as monoclonal antibodies, have been designed to block this autoimmune reaction in MS patients. However, these therapies have a limited effect in disease progression and numerous adverse side effects.

In the last years, a new strategy has emerged in order to restore self-tolerance in patients with autoimmune disorders and transplantation. This approach consists of personalized therapies using the patient’s own cells manipulated or expanded to perform tolerogenic (tolerogenic antigen-presenting cells (APC)) or regulatory functions (regulatory macrophages and T cells, Mreg and Treg).

Dendritic cells (DCs) are professional APC able to induce either immunity or tolerance. Different methodologies have been described to generate tolerogenic DCs (tolDC) ex vivo from human peripheral blood monocytes and murine bone marrow cells (pharmacological treatment, cytokines and genetic engineering) [5]. All these types of tolDC share different characteristics such as semimature phenotype—low level of costimulatory molecules and maturation-resistance— and poor stimulation of alloproliferation and induce various tolerogenic mechanisms: induction of T cell anergy, generation of Treg and/or T cells deletion [6, 7].

Our group has been working on an autologous specific tolDC therapy for MS patients for many years. We have developed a methodology to obtain myelin antigens pulsed-tolDC from MS patients using vitamin D_3_ (vitD3) in good manufacture practice (GMP) conditions [8]. With the purpose to evaluate the efficacy of the treatment, we used the animal model of MS, the experimental autoimmune encephalomyelitis (EAE). The studies performed show that the treatment with vitD3 tolDC-myelin oligodendrocyte glycoprotein (MOG)_40-55_ peptide (tolDC-MOG) in mice showing clinical signs of EAE was able to abrogate disease progression. However, the beneficial effect of the antigen-specific therapy was not stable and several administrations of cells were required [9]. The repetitive administration of tolDC implies a large number of leukapheresis and tolDC batches production (one for each administration) in GMP conditions, thus becoming an expensive and poor feasible treatment for translation of the clinic. The use of cryopreserved tolDC has been elucidated as a solution since one leukapheresis provides enough monocytes to produce large number of tolDC, which can be cryopreserved in ready-to-use aliquots. In this sense, Radhakrishnan and co-workers reported that cryopreserved DC vaccines retained in vitro and in vivo therapeutic efficacy to inhibit breast cancer growth in mice [10]. However, the process to obtain tolDC is most difficult and may be susceptible to the cryopreservation process. In this sense, cryopreserved tolDC were administrated during the clinical trial conducted by Giannoukakis in patients with type I diabetes (T1D) [11]. Although the treatment in patients was safe and well tolerated, to date, the effectiveness of frozen-specific tolDC-restoring self-tolerance has not been investigated in vivo. In the present study, we analyse the clinical efficacy of frozen vitD3 tolDC-MOG (f-tolDC-MOG) and the long-term treatment with repetitive administrations of these cells in mice with EAE. In addition, we also investigated immunotolerogenic mechanisms related with the clinical benefit following a long-term treatment with f-tolDC-MOG.

## Methods

### Animals

Female C57BL/6J mice, 8–10 weeks old, were purchased from Harlan Laboratories (Holand). The mice were housed under standard light- and climate-controlled conditions, with standard chow and water provided ad libitum.

All experiments were performed in strict accordance with EU and governmental regulations (Generalitat de Catalunya, Decret 214/97 30th July). The Ethics Committee on Animal Experimentation of the “Germans Trias i Pujol” Research Institute approved all procedures described in this study (protocol number: 5315).

### EAE induction and clinical follow-up

Mice were immunized subcutaneously with 100 μg of MOG_40-55_ (YRSPFSRVVHLYRNGK) (Institut de Recerca Biomèdica de Barcelona, IRBB, Barcelona, Spain) and emulsified (1:1) in Freund’s complete adjuvant containing 4 mg/mL of *Mycobacterium tuberculosis* (strain H37RA, Difco, Detroit, MI). In addition, mice were also injected intravenously with 250 ng of pertussis toxin (Sigma Chemical, St. Louis, MO, USA) at days 0 and 2.

All animals were weighed and examined daily for welfare and clinical signs. Clinical evaluation was performed in a blinded manner by two different observers according to the following criteria: 0, asymptomatic; 0.5, lost of distal half of tail tone; 1, lost of entire tail tone; 1.5, hind limb weakness; 2, hind limb paralysis; 2.5, hind limb paraplegia; 3, forelimb weakness; 4, quadriparesia; 4.5, severe quadriparesis; 5, quadriplegia; and 6, death. Endpoint criteria were established to minimize suffering and ensure animal welfare.

### Frozen bone marrow-derived dendritic cells

Bone marrow-derived DCs (BMDCs) were generated as previously described by Mansilla et al. 2015 [9]. Briefly, progenitor bone marrow cells were obtained from C57BL/6 donor mice and were cultured in medium containing 1000 IU/mL of granulocyte-macrophage colony-stimulating factor (GM-CSF; Prospec, Rehovot, Israel). TolDC were generated by adding 1 nM 1α,25-dihydroxyvitamin D_3_ (Calcijex, Abbott Laboratories, IL, USA) for 8 days. On day 7, 0.1 mg/mL lipopolysaccharide (LPS; Sigma) was added to the culture medium of mature DCs (mDC) and tolDC, except in the case of immature DCs (iDC). After 22–24 h, DCs were pulsed with 10 μM MOG_40-55_ (tolDC-MOG) for 18 h or cultured with only medium (unpulsed tolDC). Finally, 10 × 10^6^ tolDC or tolDC-MOG were cryopreserved in a proportion 1:1 in freezing medium containing FBS and 20 % (*v*/*v*) DMSO. The freezing medium was added drop by drop to the cells in cryovials, then transferred to containers with 2-isopropanol and maintained at −80 °C for 24–48 h. Afterwards, cells were transferred and stored in a liquid nitrogen container until use (cell aliquots remained a minimum of 4 days and a maximum of 8 months in liquid nitrogen).

### In vivo treatment with frozen tolerogenic DC

Unpulsed f-tolDC and f-tolDC-MOG were thawed in 37 °C water bath and washed twice with PBS. Cell viability and counting were calculated using Perfect-Count Microspheres (Cytognos SL), Annexin V-allophycocyanin (APC) (Immunoltools, Friesoythe, Germany) and 7-AAD (Becton Dickinson [BD] Pharmingen, San Diego, CA, USA) staining. Each mouse received a total of three doses of 10^6^ viable f-tolDC-MOG or PBS (vehicle) intravenously (iv) and spaced by a fixed period of 4 days between administrations. For the long-term treatment with frozen cells (f-tolDC-MOG, f-tolDC or PBS), the three initial doses each 4 days were followed by administrations which was required by an increase in the mean clinical score of the f-tolDC-MOG group.

### Antigen-specific T cell reactivity

Mice treated with f-tolDC-MOG or PBS (sham) on days 15, 19 and 23 pi (*n* = 8 in each group) were euthanized on day 30 pi. Splenocytes were cultured in a 96-well plate at 2 × 10^5^ cells/well in 200 μL of supplemented RPMI containing 5 μM MOG_40-55_ and either 5 μM of phorbol 12-myristate 13-acetate (PMA) plus ionomicyn (both from Sigma, positive control) or culture medium (negative control). After 48 h of culture 1 μCi/well of [3H]-thymidine (PerkinElmer, Waltham, MA, USA) was added to each well for the last 18 h of culture. The stimulation index (SI) for each stimulus was calculated as the mean counts per minute (cpm) of antigen-stimulated cultures divided by the mean cpm of the nonstimulated cultures.

### Antigen-specific proliferative stimulation of BMDC

To determine the antigen-specific stimulatory capacity of fresh and frozen iDC, mDC and tolDC pulsed with MOG_40-55_, a total of 5 × 10^3^ of viable DC were incubated for 5 days in supplemented RPMI containing 5 μg MOG_40-55_ and 10^5^ splenocytes from MOG-immunized C57BL/6 mice (ratio 1:20), adding 1 μCi/well of [3H]-methylthymidine for the last 18 h.

### Flow cytometry

Surface phenotype of fresh and frozen iDC, mDC and tolDC and phenotype of DC after 24 h stimulation with 0.1 mg/mL of LPS were determined by cellular staining with anti-CD40, CD86 and IAIE (all from BD Bioscience) and analysed in a FACSCanto II and FACSDiva software (BD).

Lymphocyte subpopulations from ex vivo splenocytes were analysed following the manufacturer’s instructions. Briefly, 0.5 × 10^6^ splenocytes were stained with mAb to study Treg (anti-CD3, CD4, CD25 and FoxP3 (all from BD Pharmingen)), Breg (anti-CD19, CD5 and CD1 (Biolegend, San Diego, CA, USA)), NK and NKT cells (anti-CD3, CD4, NKp1.1, NKp46 and CD27 (Biolegend)).

### Statistical analysis

Statistical analyses were performed using GraphPad Prism version 5.01 for Windows (La Jolla, CA, USA). Parametric and nonparametric tests were used depending on the normality distribution of the variables. To compare data from two groups, Mann–Whitney U or *t* tests were applied. When more than two groups were compared, non-parametric one-way ANOVA (Kruskal-Wallis) followed by Dunnett’s multiple comparisons tests were applied. Fisher’s exact test was used to compare qualitative variables. Differences were considered statistically significant when *P* < 0.05. Data were expressed as the mean ± standard deviation (SD) values unless otherwise stated.

## Results

### No alterations of phenotypical and functional characteristics of f-tolDC-MOG

To investigate the applicability of cryopreserved tolDC-MOG, cells were generated as previously described [9] and frozen in medium containing 10 % DMSO. After 24–48 h, f-tolDC-MOG were stored in liquid nitrogen until use.

**Fig. 1:**
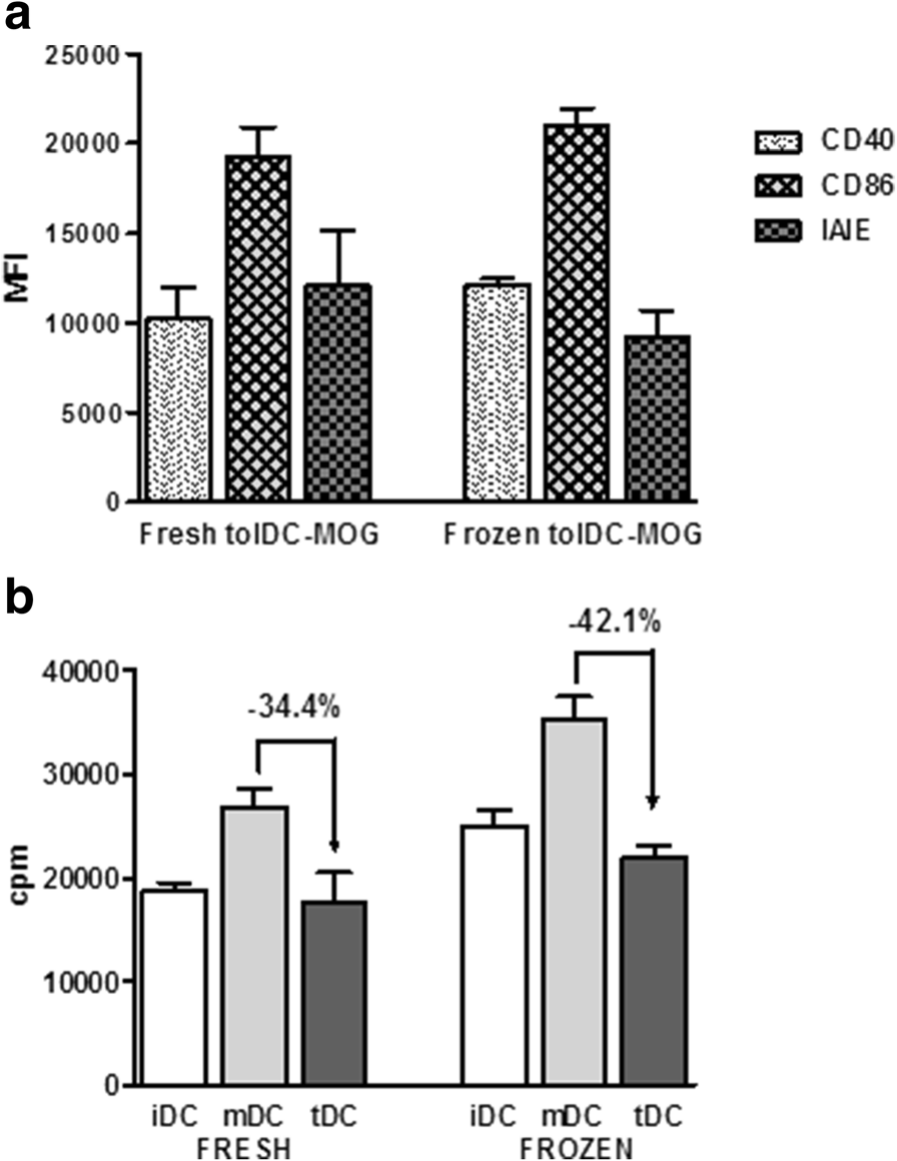
Phenotypical and functional stability of frozen tolDC-MOG. Expression of CD40, CD86 and MHCII molecule IAIE was assessed on fresh tolDC and f-tolDC-MOG. Data are expressed as mean fluorescence intensity (MFI) (**a**). Antigen-specific proliferation of MOG-reactive splenocytes co-cultured with fresh or frozen iDC-MOG, mDC-MOG or tolDC-MOG. Data are expressed as counts per minute (cpm) (**b**). Data from three independent experiments. *Error bars* correspond to SEM

Analysis of the expression of costimulatory molecules CD40 and CD86, as well as the MCH class II molecule IAIE, revealed that f-tolDC-MOG phenotype remained stable (similar level of expression of CD40, CD86 and IAIE in fresh and frozen tolDC) (Fig. 1a). In addition, f-tolDC-MOG showed a similar capacity to reduce antigen-specific T cell reactivity to fresh tolDC-MOG (Fig. 1b).

**Fig. 2:**
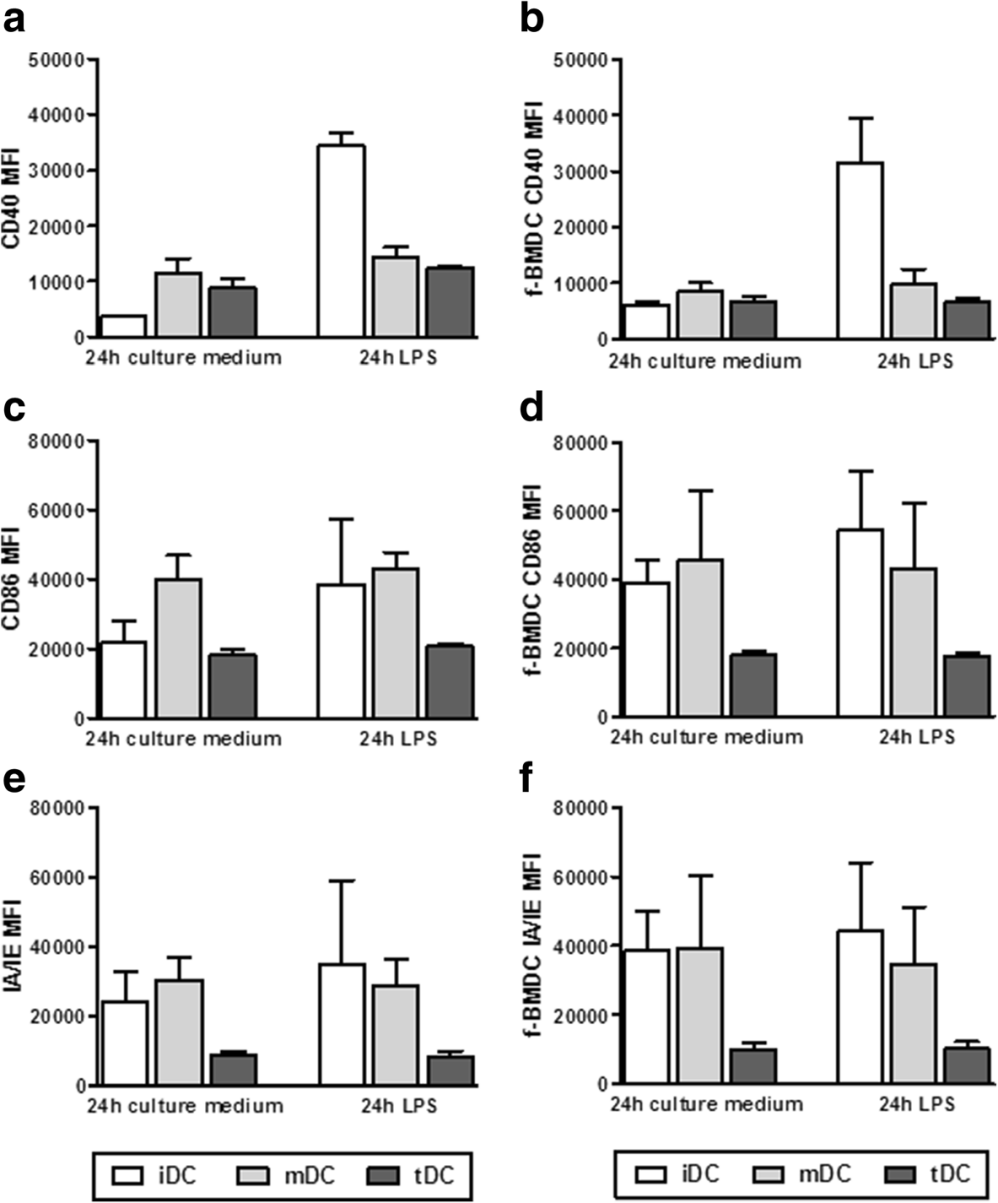
Cryopreserved tolDC-MOG are phenotypically stable following stimulation with LPS. Expression of CD40 (**a, b**), CD86 (**c, d**) and MHCII molecule IAIE (**e, f**) on fresh and frozen iDC-MOG, mDC-MOG and tolDC-MOG, after 24 h re-stimulation with LPS. Data are expressed as mean fluorescence intensity (MFI). Data correspond to three independent experiments. *Error bars* correspond to SEM

Stability of f-tolDC-MOG was assessed after the addition of a powerful inflammatory stimulus (LPS) for 24 h. In contrast to fresh or frozen iDC re-stimulated with LPS—which exhibited an increased expression of costimulatory molecules (mainly CD40) due to their maturation—both, fresh and f-tolDC-MOG showed a stable phenotype (Fig. 2).

### The treatment with cryopreserved tolDC-MOG ameliorates EAE

**Fig. 3:**
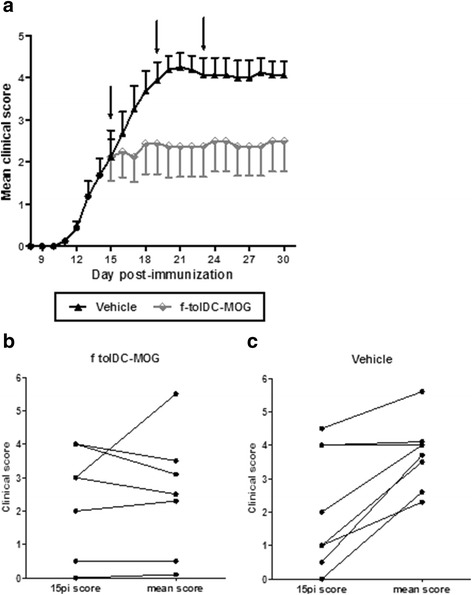
Cryopreserved tolDC-MOG treatment suppresses EAE progression. Clinical follow-up of mice treated with 1 × 10^6^ viable frozen tolDC-MOG (f-tolDC-MOG, *n* = 8) or PBS (vehicle, *n* = 8) on days +15 pi, +19 pi and +23 pi (**a**). *Arrows* indicate days of treatment administration. *Error bars* correspond to SEM. Representation of individual clinical score of the initial day of treatment (day 15 pi) and the corresponding mean clinical score during the treatment period (from 16 pi to 30 pi) from mice treated with f-tolDC-MOG (*n* = 8) (**b**) or PBS (vehicle, *n* = 8) (**c**). The mean clinical score during the treatment for each mouse was calculated as the sum of daily scores from day 16 pi to 30 pi divided by 15 (number of follow-up days after treatment)

To analyse the in vivo effect of f-tolDC-MOG, a total of 1 × 10^6^ viable f-tolDC-MOG or 200 μl PBS (vehicle) were administrated intravenously (iv) to mice showing clinical signs of EAE (on days +15, +19 and +23 pi). The treatment with f-tolDC-MOG impeded disease progression and, therefore, animals receiving f-tolDC-MOG showed an ameliorated clinical course of the disease compared to animals treated with PBS, control group (f-tolDC-MOG = 2.39 ± 0.10, PBS = 3.91 ± 0.42; *p* < 0.0001) (Fig. 3a). In accordance, maximum and cumulative scores of the group treated with f-tolDC-MOG were reduced when compared to the control group (Table 1). To determine the clinical efficacy of f-tolDC-MOG treatment to improve EAE, non-responder (NR) criteria was set up. Mice showing a ≥1 point increment in the mean clinical score compared to their respective initial clinical score (day +15 pi) were considered as NR. For that, the mean clinical score during the period of treatment duration (day +16 pi to +30 pi) was calculated for each mouse. Just only one mouse from the total of eight mice receiving f-tolDC showed a NR behaviour (12.5 % NR), indicating the relevant beneficial clinical effect of f-tolDC-MOG (*p* = 0.041) compared with the control group (75 % NR) (Fig. 3b, c).

**Table 1:** Clinical data of experimental autoimmune encephalomyelitis (EAE) mice treated with frozen tolDC-MOG (f-tolDC-MOG) and f-tolDC

	30-day follow-up		74-day follow-up		
	Vehicle	f-tolDC-MOG	Vehicle	f-tolDC	f-tolDC-MOG
Maximum score	4.25 ± 0.96	2.69 ± 2.03	4.27 ± 1.21	4.14 ± 1.22	3.13 ± 1.79
Cumulative score^a^	60.75 ± 17.92	38.00 ± 30.67	211.1 ± 83.19	188.4 ± 66.86	159.3 ± 96.58

^a^Mean of the sum of daily scores for each mouse. Data are expressed as mean ± SD

### Increment of f-tolDC-MOG therapeutic effect during a repetitive and long-term administration

**Fig. 4:**
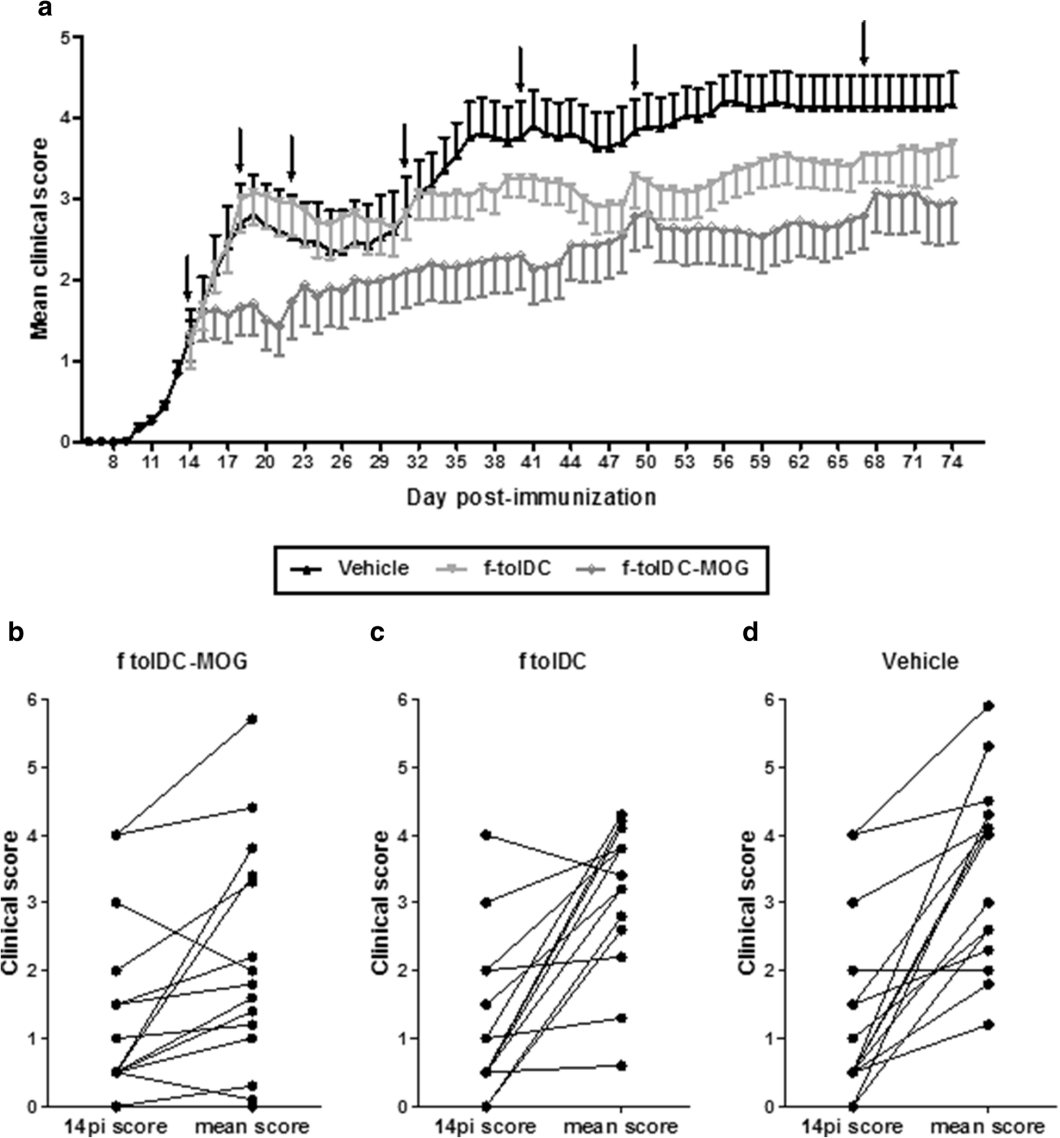
Treatment with frozen tolDC-MOG (f-tolDC-MOG) shows a long-term beneficial effect. Clinical follow-up of mice treated with 1 × 10^6^ viable f-tolDC-MOG (*n* = 15), unpulsed f-tolDC (*n* = 14) or PBS (vehicle, *n* = 15) on days +14 pi, +18 pi, +22 pi, +31 pi, +40 pi, +49 pi and +67 pi. *Arrows* indicate days of treatment administration (**a**). *Error bars* correspond to SEM. Representation of individual clinical score of the initial day of treatment (day 14 pi) and the corresponding mean clinical score during the treatment period (from 15 pi to 74 pi) from mice treated with f-tolDC-MOG (*n* = 15) (**b**), f-tolDC (*n* = 14) (**c**) or PBS (vehicle, *n* = 15) (**d**). The mean clinical score during the treatment for each mouse was calculated as the sum of daily scores from day 15 pi to 74 pi divided by 60 (number of follow-up days after treatment)

When f-tolDC-MOG treatment efficacy in EAE was demonstrated, a prolonged treatment with f-tolDC-MOG, unpulsed f-tolDC or PBS was performed to determine the long-term efficacy and tolerability of the treatment. As is shown in Fig. 4a and Table 1, the treatment with f-tolDC-MOG until day +74 pi retained a prolonged clinical beneficial effect compared to vehicle or tolDC group (f-tolDC-MOG = 2.35 ± 0.44, PBS = 3.51 ± 0.71, f-tolDC = 3.12 ± 0.37; *p* < 0.0001 in both comparisons). The repetitive and long-term administration of unpulsed f-tolDC also exhibited a slighter amelioration of EAE clinical signs compared to PBS-treated EAE mice (*p* < 0.0001) (Fig. 4a and Table 1). However, following the NR criteria, 9 out of 14 mice receiving unpulsed f-tolDC did not show clinical improvement (64.3 % NR), meanwhile only 4 out of 15 mice treated with f-tolDC-MOG were NR (26.67 % NR) (Fig. 4b, c). As a result, compared to the control group (Fig. 4d), only mice receiving f-tolDC-MOG showed a significant clinical responding behaviour (f-tolDC *p* = 0.895; f-tolDC-MOG *p* = 0.003).

Finally, it was observed that long-term treatment with unpulsed f-tolDC and f-tolDC-MOG were well tolerated and, interestingly, periods of clinical stability were progressively longer after each administration, extending the required interval dosing: first three administrations each 4 days; three administrations each 9 days, and finally, one administration after 18 days (Fig. 4a).

### Frozen tolDC-MOG treatment reduces antigen-specific response

**Fig. 5:**
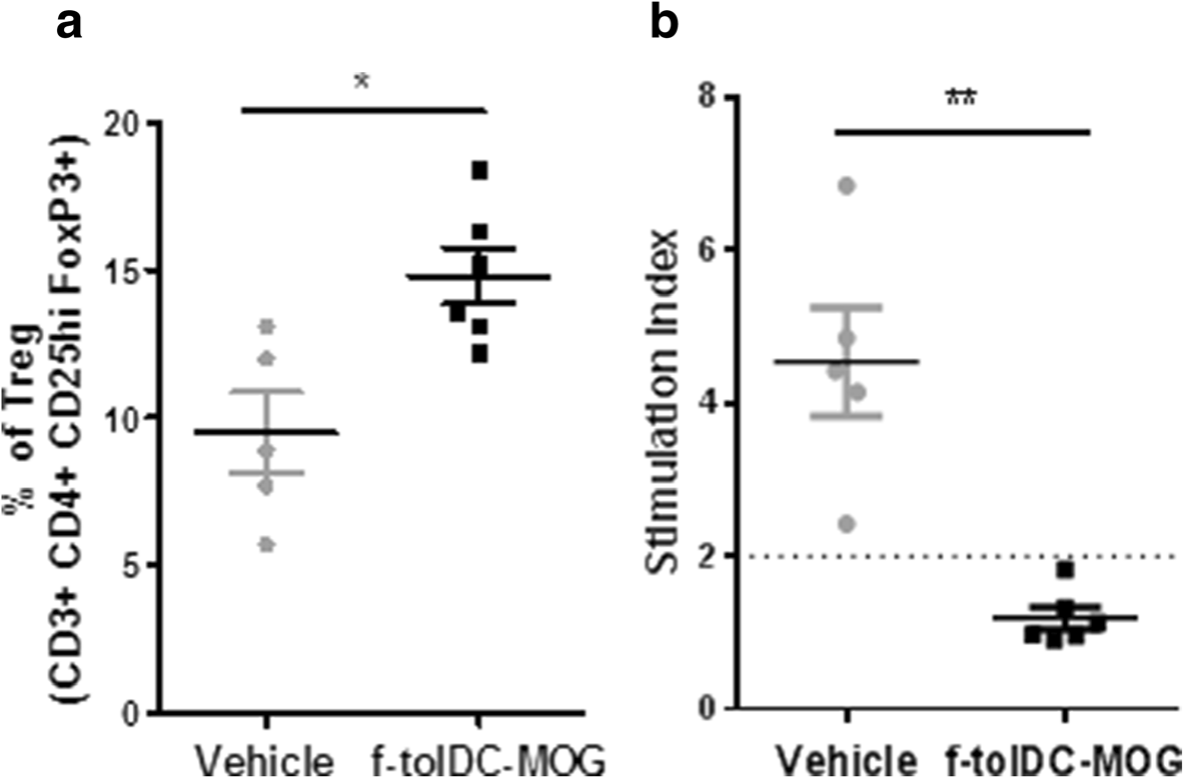
Reduced antigen-specific reactivity and increased percentage of regulatory T cells (Treg) of splenocytes from mice treated with frozen tolDC-MOG (f-tolDC-MOG). Percentage of CD25 + FoxP3+ cells (Treg) in ex vivo splenocytes of f-tolDC-MOG and PBS-treated mice (*n* = 5 and *n* = 6, respectively) (**a**). MOG-induced proliferative response from mice treated with f-tolDC-MOG (*n* = 5) or PBS (vehicle, *n* = 6). The data are expressed as stimulation index (SI). The *error bars* correspond to SEM. *p < 0.05, **p < 0.01 (**b**)

To characterize the response induced by f-tolDC-MOG, splenocytes from mice sacrificed at day 30 pi were obtained. Compared to vehicle-treated mice, splenocytes from mice treated with f-tolDC-MOG showed an increased percentage of Treg (CD3+ CD4+ CD25+ FoxP3+ cells 14.80 ± 2.30 vs. 9.48 ± 3.05, *p* = 0.017) and a reduced MOG-proliferative response (1.19 ± 0.35 vs. 4.54 ± 1.59, *p* = 0.004) (Fig. 5a, b).

### The maintenance of tolerogenicity is induced by a reduction of NK cells alongside increment of Breg and immunoregulatory NKT cell potential

**Fig. 6:**
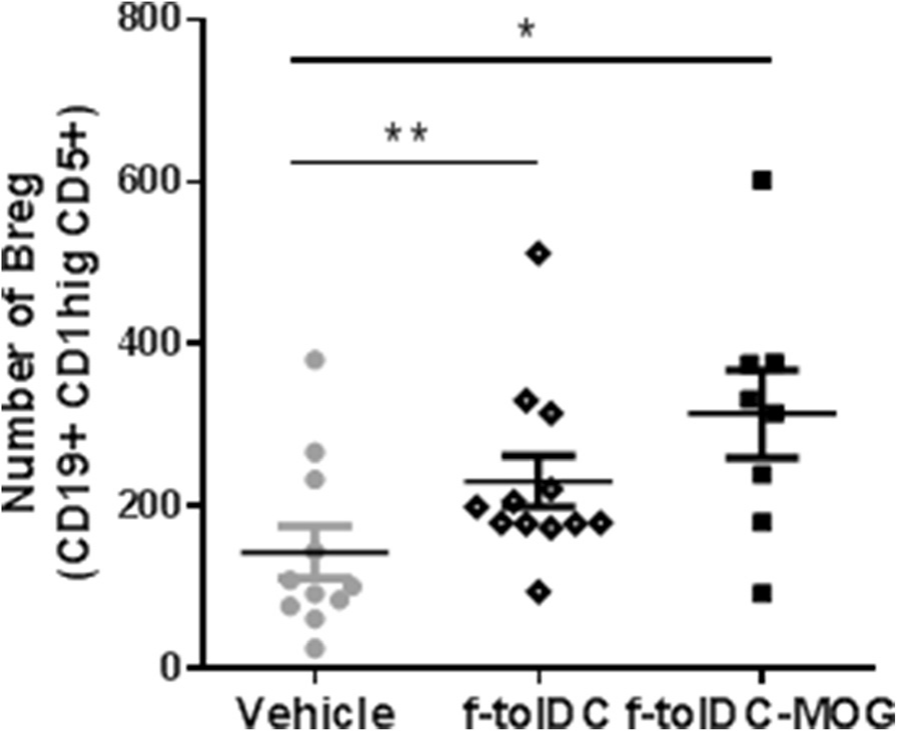
Treatment with tolDC increases regulatory B cells (Breg) in mice with EAE. Number of CD19+ CD1hi CD5+ cells (Breg) in ex vivo splenocytes of f-tolDC-MOG, f-tolDC and PBS-treated mice (*n* = 8, *n* = 12 and *n* = 11, respectively). The *error bars* correspond to SEM. *p < 0.05, **p < 0.01

**Fig. 7:**
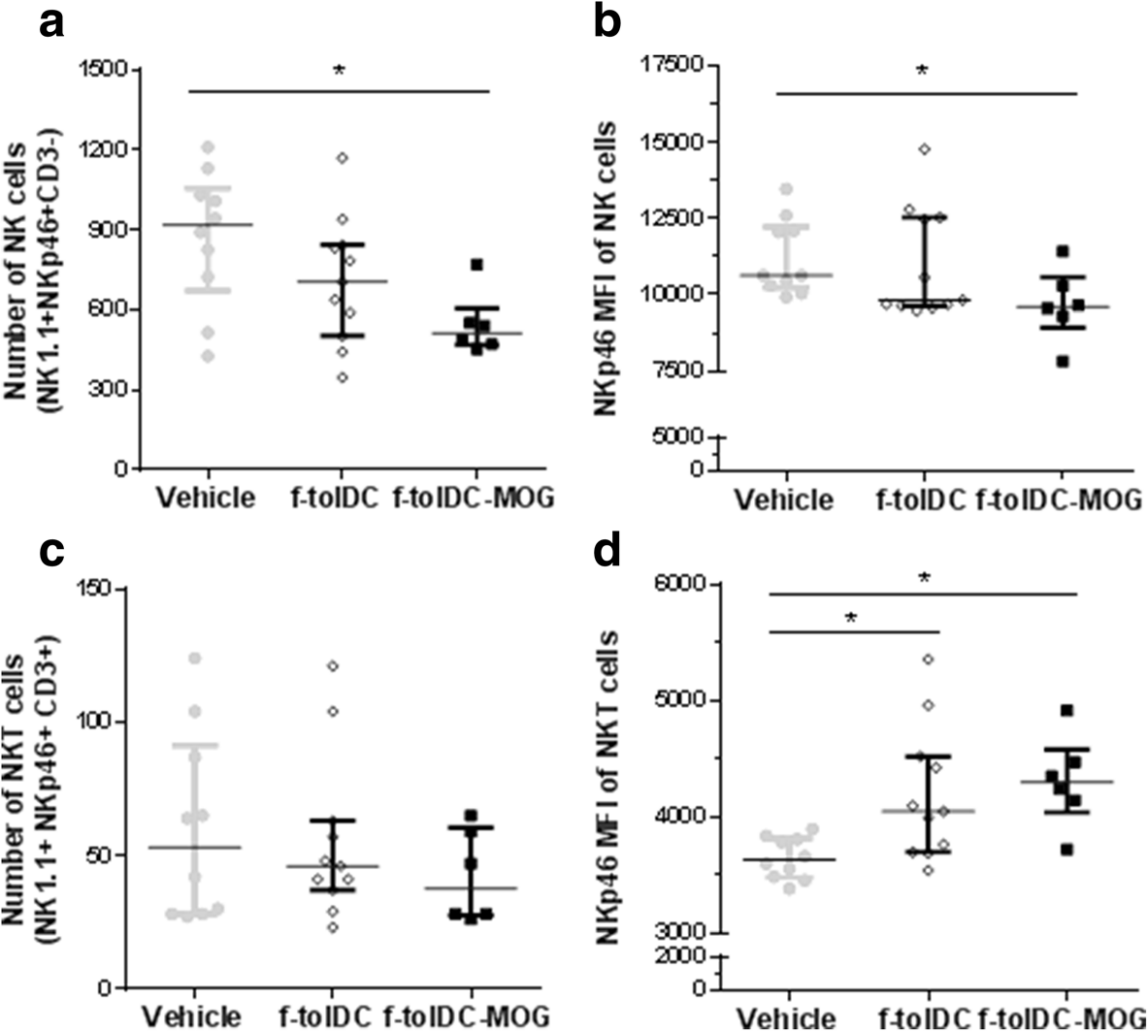
Effect of treatment with tolDC on NK and NKT cell subpopulations. Number of NK cells (NKp1.1+ NKp46+ CD3−) (**a**) and median fluorescence intensity (MFI) of the NKp46 activation marker (**b**) in ex vivo splenocytes from f-tolDC-MOG, unpulsed f-tolDC and PBS-treated mice (*n* = 6, *n* = 11 and *n* = 10, respectively). Number of NKT cells (NKp1.1+ NKp46+ CD3+) (**c**) and NKp46 MFI (**d**) of splenocytes from f-tolDC-MOG, unpulsed f-tolDC and PBS-treated mice (*n* = 6, *n* = 11 and *n* = 10, respectively). The *error bars* correspond to SEM. *p < 0.05

In addition, further immune cell populations were analysed in mice having a long-term treatment (2 months) with f-tolDC-MOG, f-tolDC or PBS. The results revealed an increased number of Breg (CD19+ CD1dhigh CD5+ cells) in mice treated with both f-tolDC-MOG and unpulsed f-tolDC compared to the vehicle group (313.8 ± 153.0 and 230.3 ± 108.9 vs. 142.4 ± 106.9; *p* = 0.039 and *p* = 0.019, respectively) (Fig. 6). When NK cells were examined (NKp1.1+ NKp46+ CD3− cells), a reduction in the number of NK cells was observed in f-tolDC-MOG and f-tolDC-treated mice, although differences with the control group were only significant in the group receiving antigen-specific cells (f-tolDC-MOG) (*p* = 0.132 and *p* = 0.031, respectively) (Fig. 7a). In the same way, the expression level of NKp46 activation marker was also reduced only in f-tolDC-MOG (f-tolDC-MOG 9677 ± 1184, f-tolDC 11,003 ± 1820 and PBS 11,218 ± 1232; *p* = 0.015 and *p* = 0.286, respectively) (Fig. 7b). Furthermore, the expression of CD27 was also analysed in the NK cell population. The tolerogenic therapy with unpulsed f-tolDC and f-tolDC-MOG reduced the number of NK CD27- and NK CD27 low cells (reaching the statistical significance only in f-tolDC-MOG-treated mice), in contrast, only mice receiving f-tolDC-MOG showed a reduction in number of NK CD27high cells (*p* = 0.045) (Table 2).

**Table 2:** Analysis of NK cells in mice receiving tolerogenic therapy

	NK CD27−	NK CD27low	NK CD27hi
f-tolDC-MOG	177.5 ± 42.1*	66.67 ± 19.3*	255.7 ± 74.7*
f-tolDC	176.7 ± 108.1*	87.09 ± 33.3	384.9 ± 144.7
Vehicle	295.9 ± 126.0	123.0 ± 42.8	391.9 ± 147.8

Data are expressed as mean ± SD

*p < 0.05

Finally, the analysis of NKT cells (NKp1.1+ NKp46+ CD3− cells) revealed no differences in the number of these cells in any treatment group (Fig. 7c). However, an increase in the expression of NKp46 in NKT cells from mice receiving a tolerogenic therapy (f-tolDC and f-tolDC-MOG) was found in comparison with the mice treated with PBS (f-tolDC-MOG 4311 ± 392.9, f-tolDC 4193 ± 573.0 and PBS 3649 ± 179.2; *p* = 0.003 and *p* = 0.010, respectively) (Fig. 7d).

## Discussion

The encouraging results obtained from different in vitro and pre-clinical studies in animal models have revealed tolDC as a promising therapy for autoimmune diseases and transplantation [5, 12, 13]. Consequently, some clinical trials using tolDC have been initiated. The first phase I clinical trial was carried on by Giannoukakis et al. in T1D patients treated with autologous tolDC generated using antisense oligonucleotides against costimulatory molecules. The study reported that tolDC treatment was safe and well tolerated, opening a new way to treat autoimmune diseases without the side effects related to immunosuppressive drugs [11]. In the same way, other clinical trials have been recently reported in other autoimmune diseases [14–16] (reviewed in [17]). However, the production of tolDC in GMP conditions is expensive and some critical points need to be solved in order to bring tolDC therapy from the bench to the bedside. The use of cryopreserved tolDC may be a most feasible strategy, because it allows producing only one batch of tolDC for all the required administrations during the treatment. Therefore, the use of f-tolDC would avoid submitting the patient to one leukapheresis process for each cell injection, thus, reducing the production cost and variability between tolDC batches. In contrast to immunogenic DC, in which their function is retained after cryopreservation [10], the in vivo beneficial effect of frozen tolDC has not been demonstrated so far.

In the present work, we have demonstrated that vitD3 f-tolDC-MOG not only show in vitro phenotypical and functional stability but also are able to abrogate EAE disease progression in vivo with clinical signs of EAE. Furthermore, the results obtained by injecting vitD3 f-tolDC-MOG were comparable to our previously published study treating EAE mice with fresh vitD3 tolDC-MOG, using the same administration schedule [9]. Together with the suppression of antigen-specific reaction and the increase of Treg response observed, results indicate that frozen and fresh tolDC-MOG exert the same beneficial mechanisms in mice with clinical signs of EAE.

Once the tolDC-MOG production was optimized using frozen cells, the next step was to analyse the effect of a long-term treatment with such cells and get further information for the translationality of this therapy to MS patients. For this purpose, an experiment of 74 days follow-up was performed. The three initial administrations were performed in a short period (every 4 days 14, 18 and 22 pi) as a shot to stop disease progression, while the next administrations were performed only when a worsening in the f-tolDC-MOG-treated group was detected. Interestingly, we observed that after several administrations of f-tolDC-MOG, periods of clinical stability were progressively longer, thus indicating that a more stable or prolonged tolerogenic effect was achieved after repetitive administrations of f-tolDC-MOG.

To determine the clinical effectiveness of the treatment with f-tolDC-MOG, classification of NR mice was established as mice showing an increment of ≥1 point in the mean clinical score during the treatment period (from 15 pi to 74 pi), compared to their respective score of the first day of treatment (14 pi). As shown, the antigen-specific therapy demonstrated to be the most tolerogenic treatment, being effective in 73.33 % of mice compared to the 35.7 % of the group of mice receiving unpulsed f-tolDC. In vitro studies performed with splenocytes confirmed that the beneficial effect of f-tolDC-MOG was related to an abrogation of antigen-specific response and Treg response induction, as previously described with fresh tolDC-MOG treatment [9].

Finally, the repetitive and long-term treatment with f-tolDC-MOG resulted in an intriguing prolonged clinical amelioration after each administration, suggesting that different tolerogenic mechanisms were elicited with the passing of time by this antigen-specific therapy. To investigate the mechanisms related with the maintenance of peripheral tolerogenicity, Treg (data not shown), Breg, NK and NKT cells immunoregulatory populations were analysed.

One of the most relevant tolerogenic mechanisms induced by tolDC therapies in addition to Treg are Breg (most B10 or IL-10 producers) since they have demonstrated immunoregulatory functions in EAE, inflammatory bowel disease and collagen-induced arthritis [18, 19]. The most relevant result from the phase I clinical trial in T1D was the increase frequency of immunosuppressive B cells [11, 20]. The ex vivo analysis of splenocytes from f-tolDC-MOG or f-tolDC-treated mice did not reveal differences in the number of Treg (data not shown). However, increased number of CD19+ CD1hi CD5+ cells (Breg) compared to the control group was found. Nevertheless, this regulatory mechanism was less potent in mice receiving f-tolDC than those receiving the antigen-specific therapy (f-tolDC-MOG).

A crucial role of NKT cells in anti-microbial and anti-cancer response, as well as preventing autoimmune diseases such as MS, T1D, lupus or RA, has been reported [21, 22]. NKT cells constitute a minority population of T cells that express NK receptors (i.e. NK1.1) and recognize glycolipids by semi-invariant CD1d-restricted αβ T-cell receptors (TCR). Therefore, they are in the interface between innate and adaptive immunity, showing important immunoregulatory properties [21]. The NKT-DC interactions can elicit either IFN-γ secretion and subsequent DC maturation, or IL-4 release, promoting tolDC differentiation [22]. In addition, reduced number of NKT cells in patients and animal models of autoimmune disorders (including MS and EAE) has been reported [23]. Notwithstanding, the regulatory potential of NKT is still on debate because CD1d or Jα18 knock-out C57Bl/6 mice did not develop spontaneous EAE, different strategies inducing or increasing NKT cells have ameliorated the disease symptoms.

The analysis of NKT population in EAE mice receiving tolDC-MOG, tolDC or PBS did not show differences in the number of NKT cells between groups. However, higher expression of the activation NK cell marker, NKp46, was found in mice treated with tolDC-MOG. Although additional studies must be performed, results could indicate that the repetitive and long-term treatment with tolDC-MOG increases NKT cells regulatory functions and contribute to prolong/maintain the clinical benefit in mice receiving the antigen-specific treatment.

Interestingly, a remarkable reduction in the number of NK cells was found in f-tolDC-MOG-treated mice compared to the control group. NK cell function is regulated by a balance of positive and negative signals. To investigate the activation status of NK cells from spleen cells of different mice, the expression of the receptors NKp46 and CD27 was determined. The results suggest that besides of NK cells reduction, the tolerogenic therapy with f-tolDC and f-tolDC-MOG decreased NK cell activation (measured by less NKp46 expression and reduced CD27low NK cells) compared to the PBS group. Nonetheless, the most potent effect was achieved exclusively by the antigen-specific therapy, probably due to the significant reduction of cytotoxic NK cells (CD27high), which have lower activation threshold than CD27low NK cells [24, 25].

Taken together, these results show that the treatment of mice showing EAE clinical signs with vitD3 f-tolDC-MOG was able to abrogate the disease mediated by an inhibition of antigen-specific reactivity, increment of Treg, Breg and activated NKT cells and reduction of NK cells. The long term-treatment with f-tolDC-MOG was well tolerated, highly effective and exhibited a prolonged clinical benefit after each administration.

## Conclusions

We have demonstrated that vitD3-frozen tolDC-MOG is a feasible cell-based therapy, with a potent immunoregulatory function in EAE and, potentially, in MS patients. The results emphasize the relevance to perform antigen-specific treatment. Therefore, efforts must be focus on identifying autoantigen peptide candidates to be loaded in tolDC according to the target autoimmune disease.
